# Lipid-lowering medications and risk of malignant melanoma: a Mendelian randomization study

**DOI:** 10.3389/fonc.2024.1408972

**Published:** 2024-06-21

**Authors:** BoWen Yang, HanYu Wang, WenYuan Song, JiuHuan Feng, ShuFang Hou

**Affiliations:** ^1^ Oncology, Dongguan Hospital of Guangzhou University of Traditional Chinese Medicine, Dongguan, Guangdong, China; ^2^ Graduate School of Guangzhou University of Traditional Chinese Medicine, Guangzhou, Guangdong, China; ^3^ Clinical Medical College, Chengdu University of Traditional Chinese Medicine, Chengdu, Sichuan, China

**Keywords:** lipid-lowering medications, malignant melanoma, cancer, Mendelian randomization, causal association, GWAS

## Abstract

**Background:**

The relationship between blood lipids, lipid-modifying medications, and cancer risk has been under investigation for some time. Recent studies suggest that lipid-lowering medications might influence melanoma outcomes, though findings remain controversial. Our study aims to clarify the potential causal relationship between lipid-lowering drugs commonly used and melanoma incidence through a comprehensive Mendelian randomization (MR) analysis.

**Methods:**

Genetic variations within an LDL-related drug target gene (LDL-cholesterol from a genome-wide association study) served as proxies for exposure to lipid-lowering drugs. We conducted a two-sample Mendelian randomization analysis using inverse variance weighting (IVW), MR-Egger, and weighted median approaches. The MR-PRESSO test and pleiotropy_test were utilized to identify and adjust for horizontal pleiotropy. Stability and reliability of the Mendelian randomization findings were assessed using the leave-one-out method, Cochran’s Q test, and funnel plot analysis. Odds ratios (OR) were employed to evaluate the causal relationship between genetic proxies of lipid-lowering drugs and melanoma risk.

**Results:**

IVW analysis revealed that HMGCR gene expression is linked to a decreased risk of melanoma [OR: 0.624(0.439–0.888); *p* = 0.008]. Conversely, PCSK9 gene expression is tied to an elevated risk of melanoma [OR: 1.233(1.026–1.484); *p* = 0.025]. No significant association was observed between NPC1L1 and melanoma.

**Conclusions:**

HMGCR inhibitors (statins) may increase melanoma risk, while PCSK9 inhibitors (evolocumab, alirocumab) could potentially decrease melanoma risk.

## Introduction

Melanoma ranks as the third most common type of skin cancer and has witnessed the fastest increase in incidence among all malignant tumors in recent years (1). Moreover, it remains the only type of cancer for which the objective of reducing mortality in the healthy population has not been met in recent years, imposing a significant economic burden on both individuals and society. In 2020, there were 325,000 new melanoma cases worldwide, with the highest incidence rates reported in Australia and New Zealand (2). The risk of developing melanoma is linked to factors such as ultraviolet exposure, skin pigmentation, and genetic predispositions (3). Research indicates that individuals with lighter skin are at a notably higher risk of melanoma compared to those with darker skin tones (4). Early-stage melanoma primarily undergoes surgical removal, boasting a five-year survival rate of 91% (5). However, the majority of diagnoses occur at intermediate or advanced stages, where options are limited and prognoses are poor (6). In terms of prevention, widespread screening is not recommended due to melanoma’s characteristics, with emphasis instead placed on educating and screening those at high risk (3). Identifying melanoma’s pathogenic factors and discovering new treatments are therefore critically important.

Lipid-lowering medications, such as statins, PCSK9 inhibitors, and NPC1L1 inhibitors, play a crucial role in preventing cardiovascular diseases. Beyond their primary use in cardiovascular prevention, these drugs also exhibit anti-inflammatory, antioxidant, immune-regulatory, neuroprotective, and anti-diabetic properties (7). Some studies have suggested that lipid-lowering drugs may be effective in cancer treatment (8). However, evidence indicates that statin use could increase melanoma risk (9). Current retrospective studies and meta-analyses have yet to achieve consensus on this issue. This may be attributed to the inherent methodological biases and small sample sizes of observational studies, as well as variability in regions, populations, baseline characteristics, and follow-up durations across different cohorts, which can obscure the relationship between study findings and conclusions. Therefore, it is essential to conduct comprehensive and rigorous research to definitively determine the causal link between lipid-lowering drugs and melanoma risk.

Mendelian randomization (MR) is a robust method that employs genetic variation as an instrumental variable (IV) to explore the association between exposure factors and diseases (10). MR analysis reduces confounding factors and reverse causality by utilizing the segregation and independent assortment of genes transmitted from parents to offspring. In scenarios devoid of pleiotropy (i.e., genetic variations influencing the disease via alternative pathways) and population stratification, MR can accurately determine disease risk (11). Furthermore, genetic variations can forecast and mirror the impact of medications on individuals. This is attributed to the fact that genetic variations, whether adjacent to or within the gene encoding a drug’s target, can regulate its expression, thereby influencing the activity of the pharmacological target. Our objective is to perform a two-sample MR analysis to assess the causal impact of genetic surrogates for lipid-lowering drugs on melanoma risk in a thorough and dependable manner, aiming also to offer novel insights into the prevention and treatment of melanoma.

## Methods

### Study design

**Figure 1:**
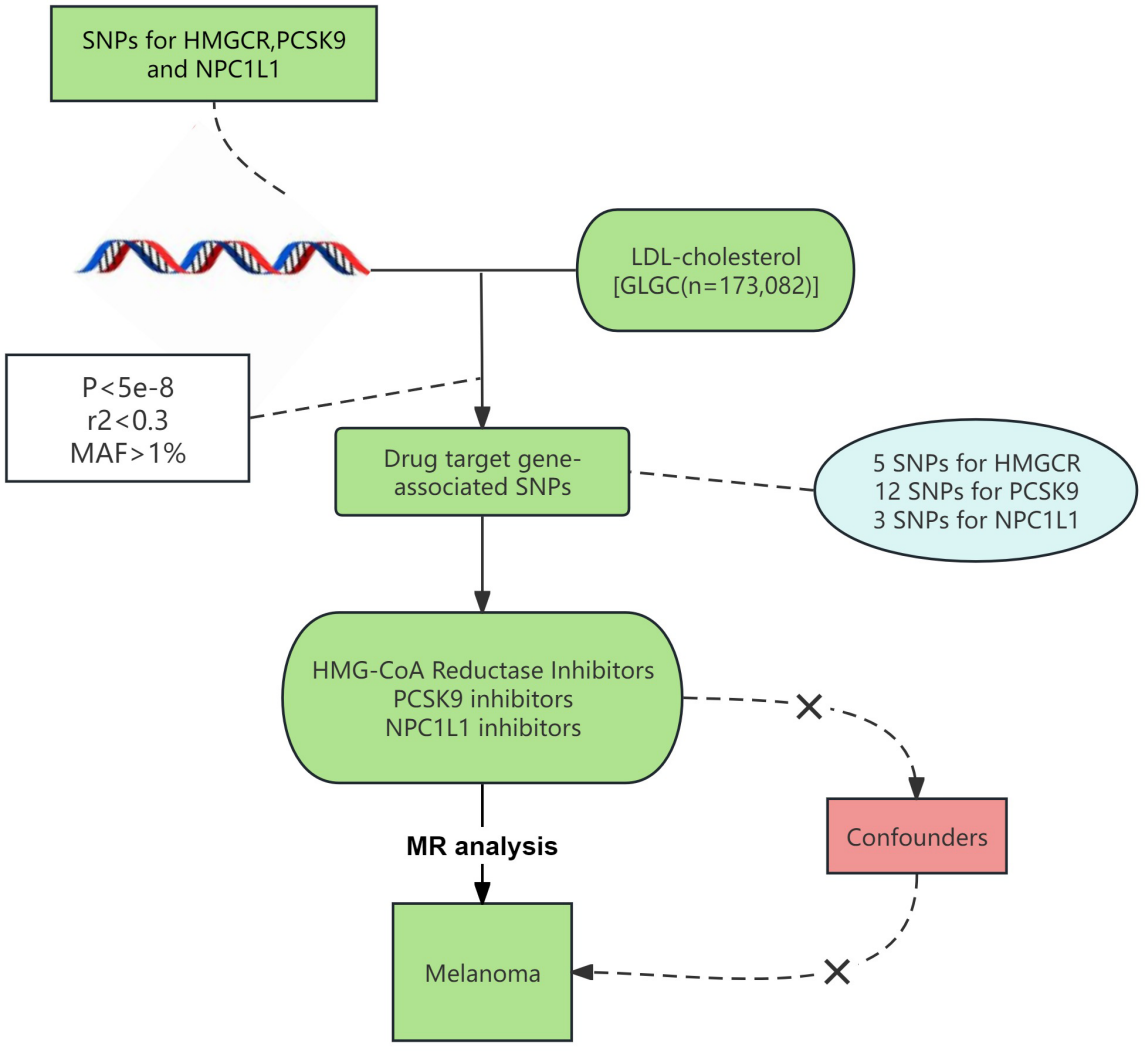
Procedure for an MR analysis of causal associations lipid-lowering medications and risk for malignant melanoma.

In **Figure 1**, we outline our MR study aimed at methodically examining the link between lipid-lowering drugs and melanoma risk. All studies included in our analysis received approval from their respective academic ethics review boards. Each participant provided written informed consent, and the necessary approvals were obtained from their institutional review committees. Notably, our research, being a reanalysis of publicly accessible GWAS data, did not necessitate further ethical approval. The study adhered to the STROBE-MR (Strengthening the Reporting of Observational Studies in Epidemiology using Mendelian Randomization) guidelines (**Supplementary Table 1**) (12).

### Data source

Aggregate genetic association data (13) were sourced from the GWAS meta-analysis of LDL-C levels conducted by the Global Lipid Genetics Consortium (GLGC). The GLGC dataset encompassed 188,577 European individuals, including 93,982 from 37 studies genotyped with the Metabochip array and 94,595 from 23 GWAS cohorts, ensuring no overlap with Metabochip cohorts (13). In the majority of the included studies, researchers opted to measure blood lipid levels in fasting blood samples (taken at least eight hours after fasting) and attempted to exclude participants on lipid-lowering medications as much as possible. Genetic instrumental variables for melanoma were obtained from the recently published R10 data bank of the FinnGen Biobank, encompassing European male and female patients (4,261 cases to 313,897 controls) (14). **Supplementary Table 2** details provides detailed information on the data sources for this study.

### Selection of instrumental variables

To ensure robust experimental outcomes, we adopted a distinctive approach to identify genetic proxies for lipid-lowering drug exposure. Initially, we selected SNPs within the 100kb vicinity of the HMGCR, PCSK9, and NPC1L1 genes. The chromosomal locations of these genes are available at the National Center for Biotechnology Information (NCBI) (https://www.ncbi.nlm.nih.gov/). Subsequently, we chose SNPs demonstrating genome-wide significance (*p* < 5.0 × 10^-8^) with LDL-C levels. Following the GWAS on lipid-lowering drugs (15, 16), we applied a relatively relaxed R^2^ threshold of 0.3 to maximize the number of IVs, thus increasing the variance explained and statistical power (15). Only common SNPs (MAF > 1%) were included as instrumental variables (17). Furthermore, we employed F statistics to verify the strength of association between IVs and exposure, with an F statistic greater than 10 considered indicative of a strong association (18). Prior to MR analysis, we undertook data harmonization procedures to ensure that the effects of SNPs on exposure and outcomes were aligned with the same allele.

### Statistical analysis

We employed the inverse variance weighting method (IVW) as our primary analytical approach to estimate causal effects (19). To corroborate the stability and direction of our findings, we additionally utilized the MR Egger (20) and Weighted Median (21) methods alongside IVW to assess causality. Heterogeneity among SNPs was gauged using Cochran’s Q test (22). In the presence of heterogeneity, the random-effects IVW model was applied; otherwise, the fixed-effects IVW model was used.

To examine the robustness of our principal findings, a suite of sensitivity analyses was conducted. Initially, the MR-Egger intercept was utilized to detect horizontal pleiotropy (20). Furthermore, MR-PRESSO (MR-PRESSO outlier test) was deployed to identify and eliminate any SNPs exhibiting significant discrepancies, thereby correcting for horizontal pleiotropy (23). Lastly, a sensitivity analysis was performed to assess the robustness of the outcomes.

Statistical analyses were conducted using the R programming language (version 4.3.1), employing the “TwoSampleMR” and “MRPRESSO” packages for MR analysis and the “forest plot” package for visualization purposes. The significance threshold was set at *p* < 0.05. The results of causal associations were presented as OR and 95% Confidence Intervals (95% CI). This study constitutes a secondary analysis of previously published data, without any alterations to the original dataset.

## Results

### Instrumental variable selection

Following the methodology outlined, we identified genetic instruments for targeting LDL-C reduction via HMGCR, PCSK9, and NPC1L1. There were 5 SNPs associated with HMGCR targets, 12 SNPs with PCSK9 targets, and 3 SNPs with NPC1L1 targets. The F statistics for these genetic instruments ranged between 71.63 and 195.81, indicating minimal instrument bias within this MR study. The details of the genetic instruments are provided in **Supplementary Table 3**.

### Results of Mendelian randomization analysis

The Cochran’s Q test revealed no heterogeneity across all MR analysis outcomes, leading to the application of the fixed effect IVW model for the main analysis (**Supplementary Table 4**). A funnel chart illustrating heterogeneity is available in **Supplementary Material**.

**Figure 2:**
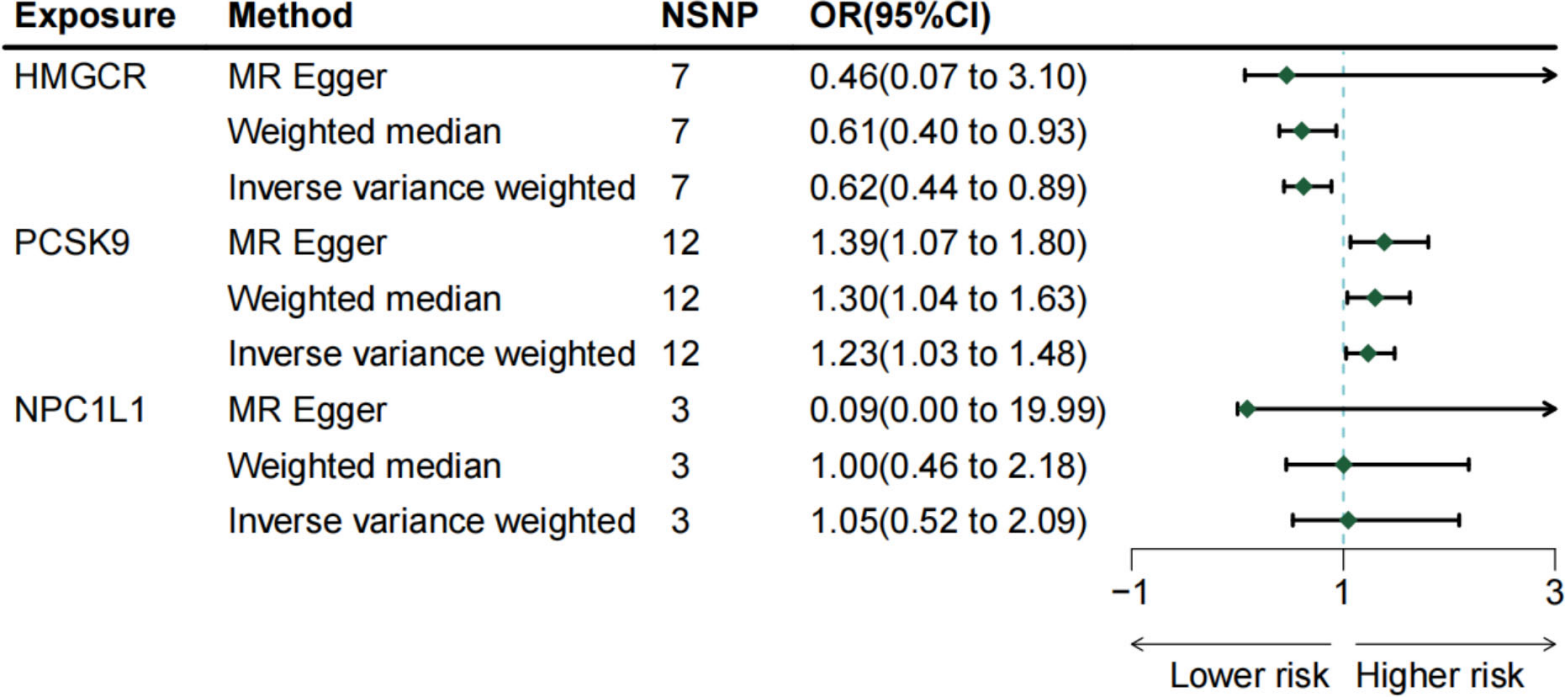
The risk association between lipid-lowering medications and malignant melanoma in a forest plot.

**Figure 3:**
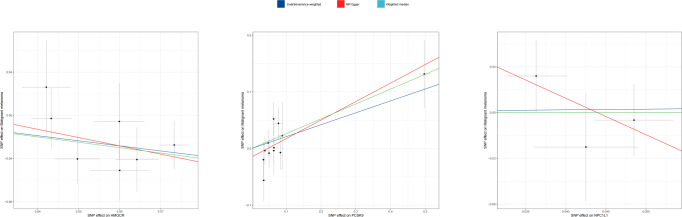
Scatter plot of MR analyses from lipid-lowering medications and malignant melanoma_revised.

Three methodologies—MR Egger, weighted median, and IVW—were employed to assess the causal connection between genetic proxies of lipid-lowering drug exposure and melanoma. IVW, the primary analysis technique, demonstrated that HMGCR expression correlates with a decreased melanoma risk [OR: 0.624 (0.439–0.888); *p* = 0.008]. The weighted median method also supported this relationship [OR: 0.607 (0.395–0.933); *p* = 0.023]. Conversely, PCSK9 gene expression was linked to an elevated melanoma risk [OR: 1.233 (1.026–1.484); *p* = 0.025]. Additionally, MR Egger [OR: 1.386 (1.067–1.802); *p* = 0.014] and weighted median [OR: 1.300 (1.038–1.628); *p* = 0.023] analyses indicated that PCSK9 expression increases melanoma risk. IVW did not demonstrate that NPC1L1 expression show a significant association with melanoma risk [OR: 0.998 (0.001–19.998); *p* = 0.899]. The primary MR analysis outcomes are depicted in **Figure 2**, with scatter plots illustrating the gene pretest effect shown in **Figure 3**.

To evaluate robustness and detect potential pleiotropy, MR-Egger intercept and MR-PRESSO tests were conducted, with *p* > 0.05, suggesting no significant bias due to pleiotropy (**Supplementary Table 4**). The leave-one-out analysis, which sequentially removes one SNP to check the consistency of the causal relationship, confirmed the stability of the MR analysis results (see **Supplementary Figure** for details).

## Discussion

To our knowledge, this is the inaugural utilization of drug target MR analysis for examining the causal link between lipid-lowering drug exposure and melanoma risk. Employing Mendelian randomization, we investigated the impact of three prevalent LDL-C reduction drug targets (HMGCR, PCSK9, and NPC1L1 genes) on melanoma risk. Our findings revealed that HMGCR was associated with a reduced risk of melanoma, PCSK9 with an elevated risk, and NPC1L1 expression bore no significant relationship with melanoma risk.

The genetic proxy for HMGCR expression is notably linked to a reduced melanoma risk, suggesting that HMG-CoA reductase inhibitors may elevate melanoma incidence. Statins, emblematic of HMG-CoA reductase inhibitors, decrease LDL-C synthesis by suppressing HMGCR expression and are the primary choice for cardiovascular disease prevention in clinical settings. A meta-analysis encompassing 11 studies observed an increased melanoma risk with statin use (9), aligning with our results. We propose that the immunoregulatory effects of statins, particularly immunosuppression, play a crucial role in this association. Research indicates increased melanoma risk in individuals experiencing immunosuppression, such as those undergoing solid organ transplants or receiving immunosuppressive therapy, compared to their non-immunosuppressed counterparts (24). Currently, it is understood that statins increase Foxp3 mRNA transcription and Foxp3 protein production by stimulating Foxp3 expression. This activation increases the proportion of regulatory T cells, leading to immunosuppression and potentially facilitating melanoma development (25). Therefore, clinical practitioners should exercise caution when prescribing statins to patients with melanoma. Moreover, vigilance against melanoma development may be warranted in patients taking Atorvastatin.

Our investigation revealed an association between PCSK9 gene expression and an increased risk of melanoma, suggesting that PCSK9 inhibitors, which reduce LDL-C levels, may offer a specific protective effect against melanoma. Despite the long-term use of PCSK9 inhibitors for managing LDL-C levels, their relationship with melanoma has been sparsely researched. Existing studies highlight the significant role of PCSK9 expression in melanoma’s pathogenesis, particularly noting the critical contribution of cholesterol accumulation in tumors facilitated by PCSK9. Cholesterol’s vital roles in cell proliferation (26) and its influence on cancer progression and the tumor microenvironment (27) are well documented. According to Yan Gu et al., PCSK9 potentially promotes melanoma partly through its regulation of lipid/cholesterol metabolism (28). Moreover, PCSK9 expression appears to have a profound impact on the systemic immune system, aiding in the immune evasion of melanoma cells. It disrupts the recirculation of major histocompatibility protein class I (MHCI) to the cell surface by binding to MHCI and facilitating its lysosomal degradation, thereby hampering the infiltration of cytotoxic T cells within tumors (29). Another study suggests the systemic influence of PCSK9 on immune mechanisms might surpass its effects on MHCI, with observed changes in the immune system including modifications to CD8 molecules, crucial for the functions of CD8+ T cells such as granzymes and perforin 1, CD3 subunits of the T-cell receptor complex (CD3E, CD3D, CD3G), complements, PAMPs like TLR8/toll-like receptor 8, cytokines (TGFβ1 and INFγ), chemokines (CCL5, CCR7, CCL4, CXCR6, among others), PI3KCG [signaling that regulates immune checkpoint PD-L1 expression in cancer cells (30)], and various immune checkpoints (PD1, PD-L1, PD-L2, CTLA4, TIGIT, CD96, LAG3, among others) (28). Additionally, Ahnak’s role in regulating melanoma metastasis to lung epithelial cells via PCSK9 expression has been identified, where PCSK9 acts as an inhibitor of TNF α-mediated apoptosis, thus promoting melanoma cell metastasis to the lungs (31). Huimin Sun et al. discovered that PCSK9 inhibitors could decrease liver metastasis of melanoma cells by reducing circulating cholesterol levels (32).

This study presents several significant advantages. Firstly, to our knowledge, this is the inaugural MR study to investigate the causal impact of lipid-lowering drugs on melanoma. Secondly, by utilizing inherited mutations in genes encoding drug targets, the study mirrors the potential effects of common LDL-C reduction therapies, thereby reflecting the influence of lifetime drug exposure on melanoma while circumventing the limitations associated with exposure and follow-up in clinical trials or observational studies. Thirdly, the employment of aggregate genetic association data from large-scale GWAS allows for causal inferences to be made with high statistical power. Fourthly, utilizing the genetic and socio-cultural homogeneity of the European population minimizes false associations due to demographic stratification and other confounding factors.

However, there are limitations to this study. The MR estimated values represent long-term regulation of disease risk by drug targets, which may not directly correlate with the effects observed during shorter periods of drug administration. Thus, the risk reduction through PCSK9 inhibitors for treating melanoma might not align with the magnitudes estimated in this MR study. Secondly, while MR analysis facilitates causal inferences, it does not explore specific biological mechanisms. Thirdly, the reliance on data predominantly from European populations limits the generalizability of the findings to other ethnic groups, necessitating future research to include diverse populations, such as Asians. Lastly, the absence of stratified analysis in the GWAS data, based on gender, age, disease progression, and other factors, restricts the ability to examine specific details, highlighting the need for future studies with larger sample sizes and more comprehensive analyses.

## Conclusion

In conclusion, through Mendelian randomization, our study explored the potential causal link between lipid-lowering drugs and melanoma. The findings indicate that HMGCR inhibitors(statins) might contribute to melanoma development, whereas PCSK9 inhibitors (iromazumab and aliciumab) could offer protective effects against melanoma. This highlights the need for cautious use of statins in the clinical management of melanoma. PCSK9 inhibitors emerge as potential protective agents against melanoma, highlighting their promise as therapeutic options. Nonetheless, the intricate relationship between lipid-lowering drugs and melanoma necessitates further clinical investigations to explore the underlying mechanisms and confirm their clinical relevance comprehensively. Thus, our study establishes a groundwork for future research in this critical area of study.

## Data availability statement

The original contributions presented in the study are included in the article/**Supplementary Material**. Further inquiries can be directed to the corresponding author.

## Author contributions

BY: Conceptualization, Data curation, Formal analysis, Funding acquisition, Investigation, Methodology, Project administration, Resources, Software, Validation, Visualization, Writing – original draft, Writing – review & editing. HW: Conceptualization, Data curation, Formal analysis, Investigation, Methodology, Project administration, Software, Writing – original draft, Writing – review & editing. WS: Formal analysis, Investigation, Writing – original draft. JF: Conceptualization, Formal analysis, Funding acquisition, Investigation, Methodology, Resources, Supervision, Visualization, Writing – review & editing. SH: Formal analysis, Funding acquisition, Resources, Supervision, Validation, Writing – review & editing.
