# Supplementary material for: Lipid-lowering medications and risk of malignant melanoma: a Mendelian randomization study
Source: Front Oncol. 2024 Jun 21;14:1408972. doi: 10.3389/fonc.2024.1408972 (PMC11224289; doi:10.3389/fonc.2024.1408972)

Supplementary Material

**Lipid-lowering Medications and Risk of Malignant Melanoma: a Mendelian Randomization Study**

**BoWen Yang^1^**^†^**, HanYu Wang^2^**^†^**, WenYuan Song^1^, JiuHuan Feng^3^, ShuFang Hou^3*^**

^1^Graduate School of Guangzhou University of Traditional Chinese Medicine, Guangzhou, Guangdong, China.

^2^Clinical Medical College, Chengdu University of Traditional Chinese Medicine, Chengdu, Sichuan, China

^3^Oncology, Dongguan Hospital of Guangzhou University of Traditional Chinese Medicine, Dongguan, Guangdong, China

*** Correspondence:**

ShuFang Hou ^1*^

[sfhoudgzyy@163.com](mailto:sfhoudgzyy@163.com)

BoWen Yang^1†^ and HanYu Wang^2†^ These authors contributed equally to this work and share first authorship.

**Supplementary Figure 1.** Leave-one-out plot and Funnel plot of MR analyses from HMGCR to Malignant melanoma

**
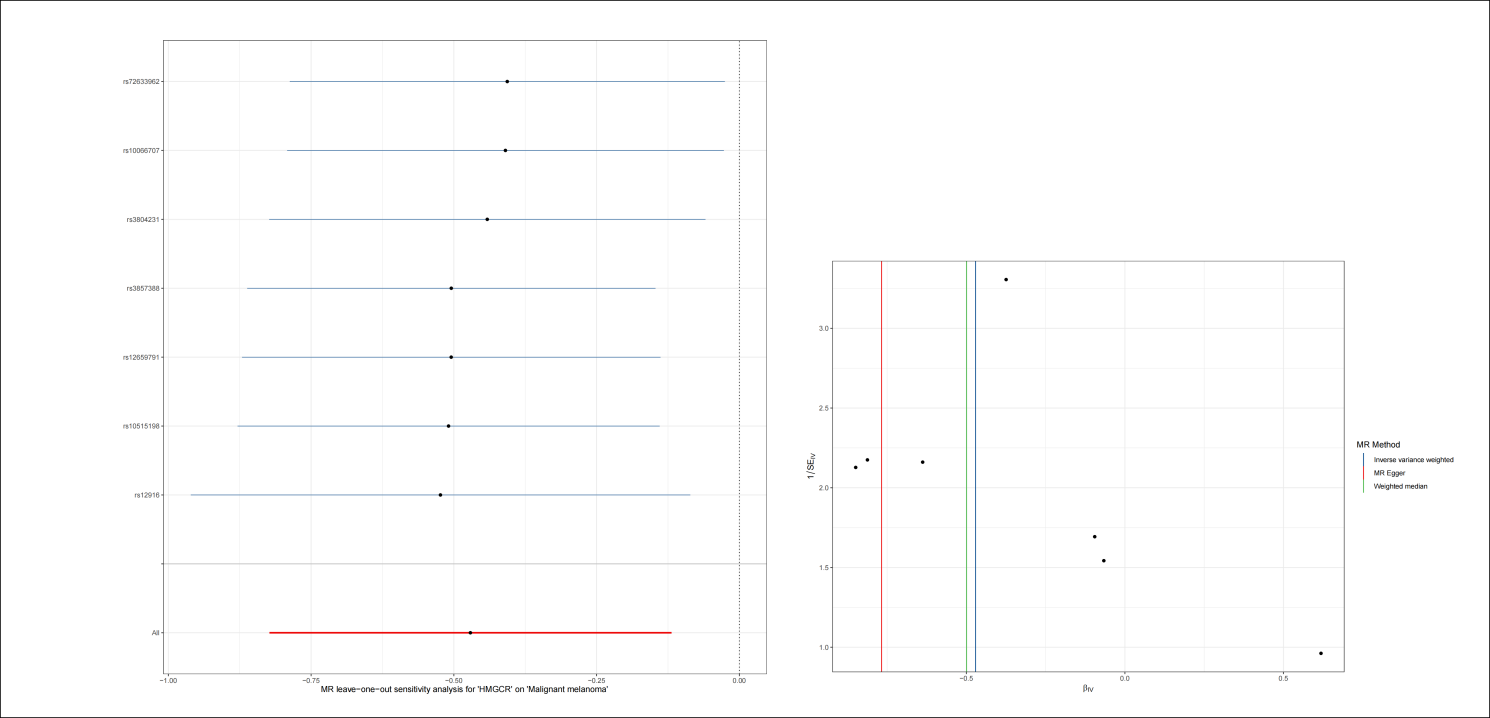
**

**Supplementary Figure 2.** Leave-one-out plot and Funnel plot of MR analyses from PCSK9 to Malignant melanoma


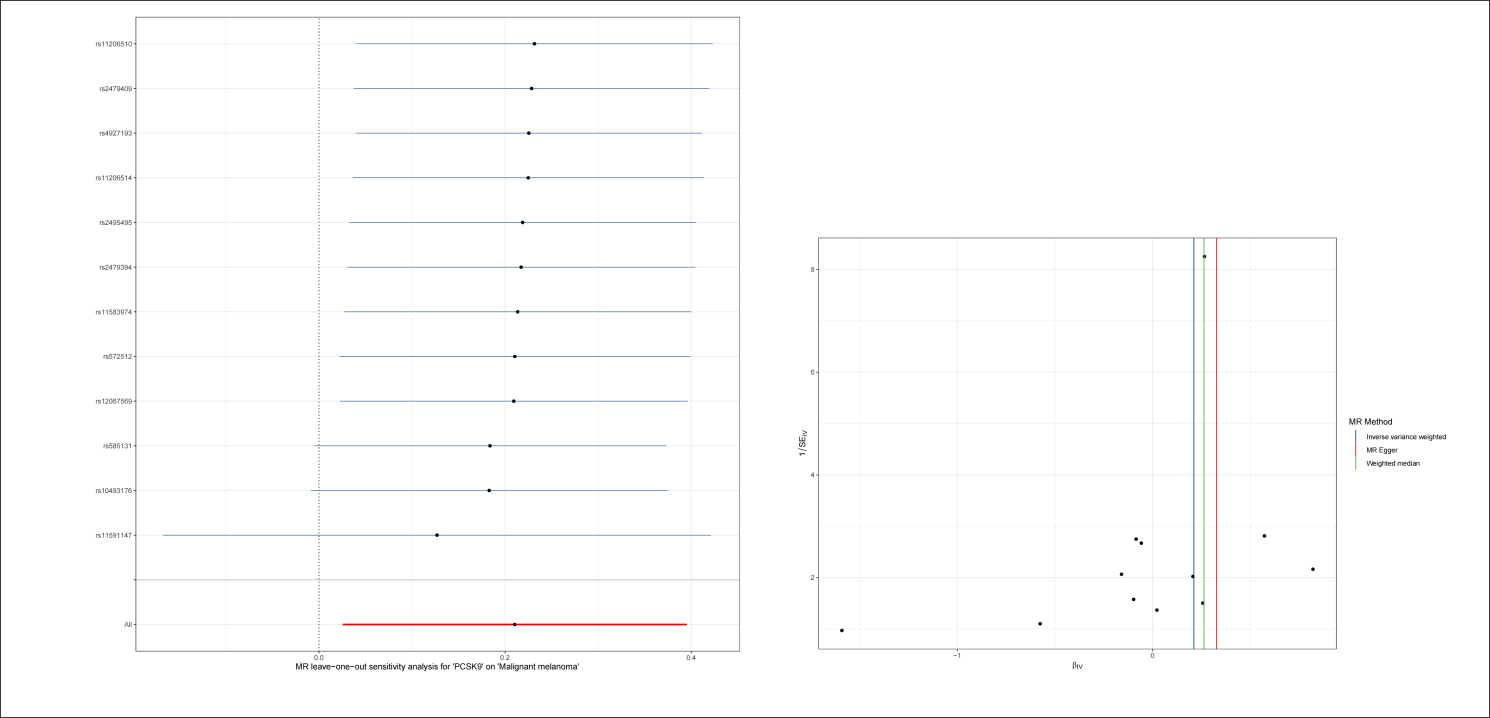


**Supplementary Figure 3.** Leave-one-out plot and Funnel plot of MR analyses from NPC1L1 to Malignant melanoma


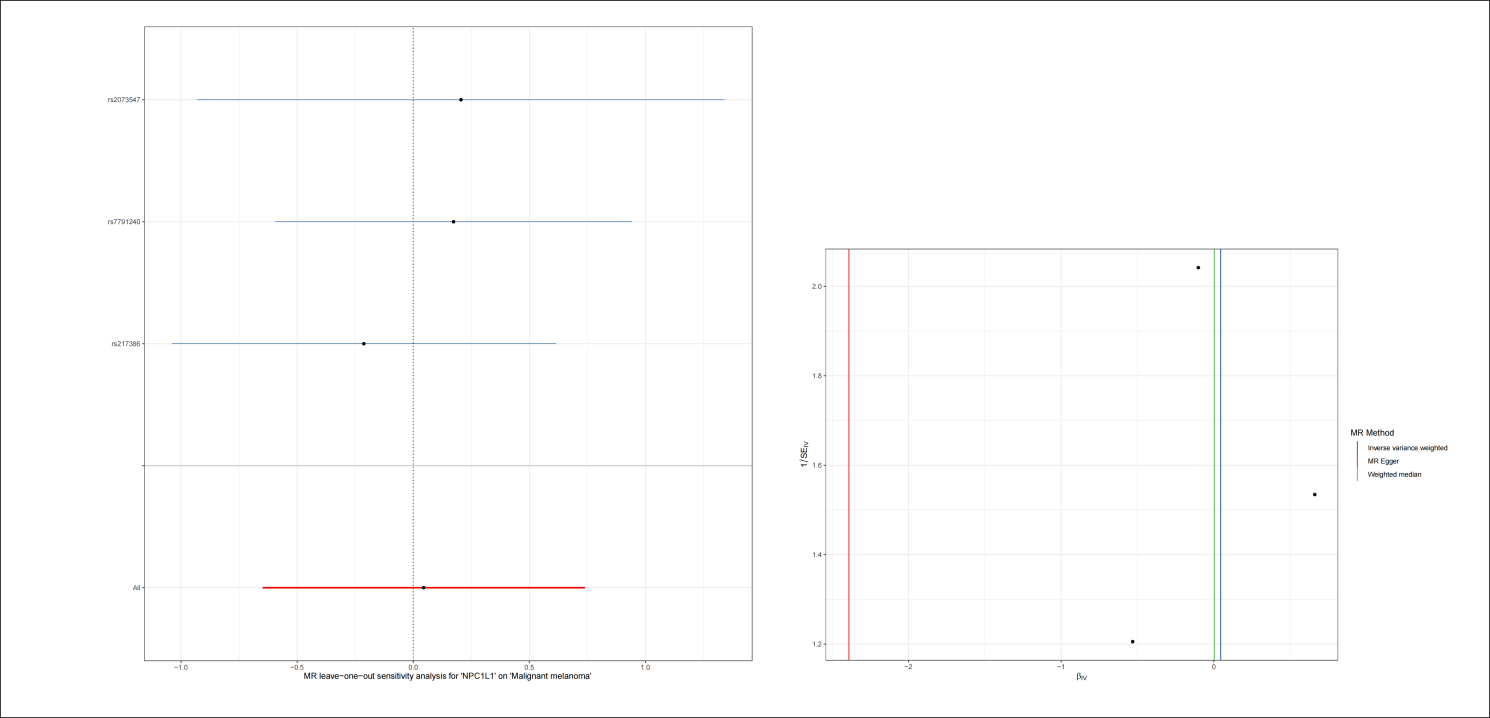

Supplement: Supplementary file 1 [file DataSheet_1.docx]
